# In Vivo and Bioinformatics Evaluation of Nine Traditional Chinese Medicine Compounds Targeting Acetylcholinesterase and Butyrylcholinesterase Enzymes in Alzheimer’s Disease

**DOI:** 10.5812/ijpr-159760

**Published:** 2025-05-04

**Authors:** Xinyu Yang

**Affiliations:** 1 School of Public Health, Inner Mongolia Minzu University, Tongliao, China

**Keywords:** Alzheimer’s Disease, Acetylcholinesterase, Butyrylcholinesterase, Oxidative Stress, Traditional Chinese Medicine, Norwogonin, Hainanolidol, Molecular Docking, Pharmacokinetics

## Abstract

**Background:**

Alzheimer’s disease (AD) is characterized by cholinergic dysfunction and oxidative stress, creating a need for the development of new therapeutic agents. Traditional Chinese medicine (TCM) compounds, such as hainanolidol and norwogonin, have shown potential neuroprotective activity.

**Objectives:**

This study investigates the inhibitory action of these compounds on cholinesterase enzymes and their possible therapeutic application in an AD-like rat model generated by lead acetate (PbAc), a well-known neurotoxicant that mimics AD-associated oxidative damage and cognitive decline.

**Methods:**

The cholinesterase inhibitory activity of nine TCM compounds was assessed in vitro against acetylcholinesterase (AChE) and butyrylcholinesterase (BuChE), with donepezil and tacrine as controls. Hainanolidol and norwogonin, identified as the most potent inhibitors, were further evaluated in a PbAc-induced AD rat model to assess their neuroprotective effects. Oxidative stress biomarkers malondialdehyde (MDA), glutathione (GSH), cholinesterase activity, and in silico molecular interactions were analyzed, including docking studies, molecular dynamics (MD) simulations, and ADME-toxicity profiling.

**Results:**

Hainanolidol and norwogonin showed strong nanomolar-range inhibition of both AChE and BuChE, with considerable IC_50_ values superior to those of standard inhibitors. In the PbAc-induced AD model, both compounds significantly reduced MDA levels and increased GSH levels, indicating oxidative stress mitigation.

The level of cholinesterase activity inhibition was as effective as, or more effective than, the suppression achieved by standard treatments, particularly regarding AChE, thus suggesting enhanced therapeutic potential compared to donepezil. Molecular docking and MD simulations confirmed stable binding interactions with key catalytic residues of AChE and BuChE, reinforcing their inhibitory mechanisms. ADME-toxicity analysis further demonstrated favorable pharmacokinetics and safety profiles.

**Conclusions:**

This study concludes that both hainanolidol and norwogonin are worthy of being regarded as dual cholinesterase inhibitors with antioxidant properties, which may serve as alternative therapeutics for AD. The use of PbAc as an AD model underscores the role of environmental neurotoxins in disease pathogenesis, offering insights into novel intervention strategies. Advanced preclinical and clinical studies are needed for further validation.

## 1. Background

Alzheimer's disease (AD) is a progressive and deadly neurological condition that presents a global health problem, especially as the population ages. Cognitive decline, including difficulties in learning, reasoning, problem-solving, memory impairment, and behavioral abnormalities like mood swings, agitation, and social disengagement, are its main symptoms (1). The AD can be diagnosed through extensive neuroinflammation, synapse loss, and neuronal death, which are defining features that cause severe cognitive and functional deficits (2). The disease is driven by numerous enzyme targets controlling these pathogenic pathways. The memory and learning neurotransmitter acetylcholine is degraded by acetylcholinesterase (AChE), resulting in an impairment of its function. The AChE inhibition leads to an increase in cholinergic signaling, which transiently assists the cognition of patients suffering from AD (3). Amyloidogenic processes involve beta secretase (BACE1), which cleaves APP to form amyloid-beta (Aβ) peptides (4). Increased BACE1 activity leads to increased Aβ accumulation, forming plaques and damaging neurons. The GSK3β enzyme is involved in tau hyperphosphorylation and neurofibrillary tangle (NFT) formation. Despite decades of research, there are limited treatments, highlighting the need for novel interventions targeting AD's complex etiology (5). Traditional Chinese medicine (TCM) is a crucial research area AD due to its complex herbal formulations containing bioactive components (6). These formulations can modify multiple cellular pathways and address various etiologies of AD, including tau hyperphosphorylation, oxidative stress, neuroinflammation, and neurotransmitter dysregulation (7). The TCM herbs like *Ginkgo biloba*, *Panax ginseng*, and *Curcuma longa* exhibit neuroprotective, anti-inflammatory, and antioxidant activities (8).

However, the precise biochemical mechanisms by which these substances affect enzyme targets in AD are underexplored. Moreover, there are numerous reports of compounds such as curcumin (*C.* *longa*), huperzine a (*Huperzia* *serrata*), berberine (*Coptis* *chinensis*), resveratrol (*Polygonum* *cuspidatum*), baicalein (*Scutellaria* *baicalensis*), and astragalosides (*Astragalus* *membranaceus*) (9).

This study investigates the molecular pathways of TCM compounds influencing AD enzyme targets using advanced bioinformatics methodologies. It aims to enhance therapeutic effectiveness by identifying synergistic interactions and assessing neuroprotective benefits in vivo using animal models. Bioinformatics methods will verify the interaction between TCM drugs and enzymes.

## 2. Objectives

This study seeks to enhance the understanding of TCM’s role in AD treatment by merging experimental and computational approaches, thus supporting its use as an adjuvant therapy for this debilitating condition.

## 3. Methods

### 3.1. Ethical Considerations

All experimental procedures involving the animal study and rats were approved by the Institutional Animal Ethics and Care Committee of Inner Mongolia Minzu University (approval No: SR-A-31/2A). The study adhered to the highest ethical standards and guidelines for animal welfare, ensuring that all procedures minimized animal distress and pain.

### 3.2. Chemicals and Reagents

All nine TCM compounds were purchased from Chengdu Biopurify Phytochemicals Ltd. (China) and Beijing Biolead International Trading Co., Ltd. (China). Donepezil and tacrine were purchased from Sigma-Aldrich, Germany. Each compound was provided with a certificate of analysis (CoA) ensuring ≥ 98% purity, which was verified by high-performance liquid chromatography (HPLC). The compounds were stored at -20°C in airtight conditions to prevent degradation. The QuantiChrom^TM^ AChE inhibitor screening kit (IACE-100) was purchased from bioassay systems, USA, and the butyrylcholinesterase (BuChE) activity kit (catalog # MBS846801) was purchased from MyBiosource, CA, and USA.

### 3.3. In Vitro Cholinesterases Inhibition and Selectivity Assay

The in vitro AChE and BuChE inhibitory effects and selectivity of nine TCM compounds were evaluated using the QuantiChrom^TM^ AChE inhibitor screening kit (IACE-100, bioassay systems, USA) for AChE inhibition and the BuChE activity kit (catalog # MBS846801, MyBiosource, CA, USA) for BuChE inhibition. For comparison purposes, clinically approved inhibitors — donepezil and tacrine — were used. The QuantiChrom^TM^ kit was used to assess the activity of AChE and BuChE by recording enzymatic activity at different concentrations of the test compounds and comparing it to activity without inhibitors, followed by plotting dose-response curves. The experimental data were fitted by nonlinear regression analysis to yield IC_50_ values. The selectivity of the compounds for AChE relative to BuChE was determined by calculating the Selectivity Index (SI) using the formula BuChE IC_50_ values are divided by AChE IC_50_ values to obtain SI.

### 3.4. In Vivo Evaluation on Rat Model of Alzheimer’s Disease

Fifty male Wistar rats (n = 50) were obtained from a certified animal facility, Shenzhen Bay Laboratory (SZBL) Laboratory Animal Platform, China. The rats were acclimatized to the laboratory environment for one week prior to the experiment. During this period, they were kept in standard polypropylene cages for rodents under controlled conditions, with a room temperature of 22°C, relative humidity of 50%, and a 12-hour light-dark cycle, with lights on from 06:00 to 18:00. The rats had ad libitum access to a standard rodent diet and filtered water to ensure optimal health and hydration. Routine monitoring of housing conditions was conducted to ensure compliance with ethical standards. To establish an AD model, the rats underwent intraperitoneal (IP) administration with lead acetate-induced AD (LA-AD, 50 mg/kg) for 15 days to mimic the neurodegenerative features of AD.

Following AD induction, the rats were randomly divided into five groups: (1) Group I (control): DMSO contained in drinking water at a 5% concentration, without neurotoxic effects, in normal rats; (2) group II (LA-AD): The LA-AD rats for 15 days; (3) group III (LA-AD-donepezil): Donepezil (1 mg/kg, IP) administered for 15 days after neurotoxicity induction in LA-AD rats. Donepezil is a widely used and well-established AChE inhibitor, and its effective dose ranges from 0.5 to 2 mg/kg, with 1 mg/kg being the optimal dose; (4) group IV (LA-AD-norwogonin): Norwogonin (1 mg/kg, IP) administered to LA-AD rats for 15 days after neurotoxicity induction. The 1 mg/kg dosage was chosen based on data available in NCBI PubChem, where flavonoids with similar chemical structures, such as baicalein, exhibited neuroprotective effects at this concentration.

(5) Group V (LA-AD-hainanolidol): Hainanolidol (1 mg/kg, IP) administered for 15 days after neurotoxicity induction in LA-AD rats. The selected dose of 1 mg/kg was based on data and studies using structurally related terpenoids, such as ginsenosides and curcumin analogs, from the NCBI PubChem Database, where the dosage ranges between 0.5 - 2 mg/kg, and 1 mg/kg demonstrated the most optimal neuroprotective effects without adverse effects.

**Figure 1.**
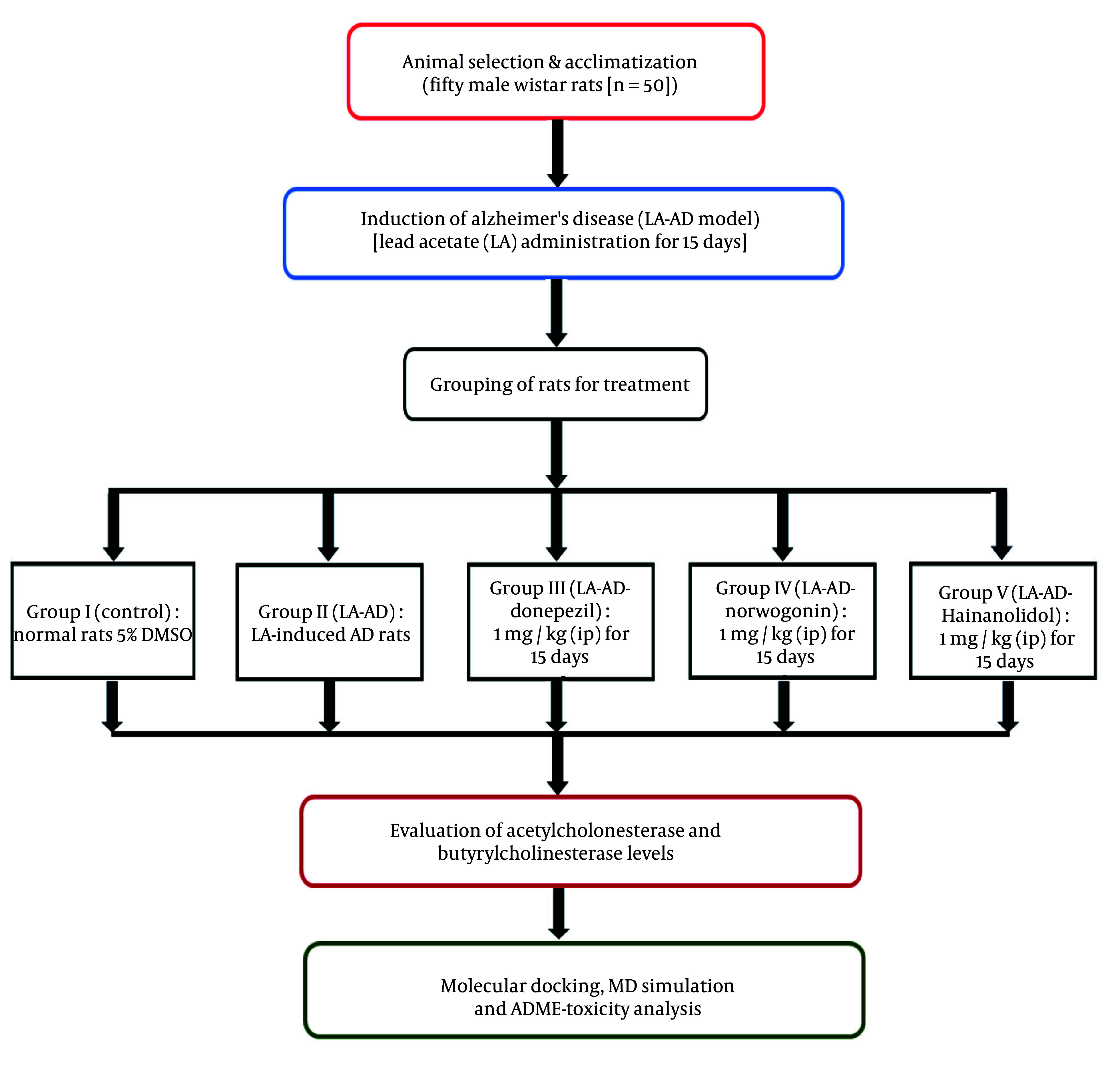
Schematic illustration representing the model construction process and the corresponding treatment regimen.

The schematic illustration depicting the model creation process and the treatment regimen is presented in Figure 1. 

### 3.5. Tissue Extraction and Blood Sampling Techniques

A 12-hour fasting period was imposed on the rats immediately after the final dose of treatment to minimize the effect of recent food intake on blood parameters. After fasting, the rats were anesthetized with a sodium pentothal solution (40 mg/kg, intraperitoneally), and blood samples were collected from the retroorbital plexus (10). The toxicological effects of the treatment were assessed through analysis of blood lead concentrations. Following 28 days of exposure, the rats were euthanized by cervical decapitation, and their brains were removed to minimize tissue damage. The brains were dissected into specific regions, including the cerebellum and cerebral cortex, for deeper analysis. The cerebellum, cerebral cortex, and hippocampus were isolated in sodium chloride solution and washed with ice-cold solution. The tissues were then homogenized, processed for biochemical analysis, and submerged in ice-cold 0.1 M phosphate buffer. Further assays using these samples were performed to evaluate biomarkers associated with oxidative stress and neurotransmitter activity.

### 3.6. Assessment of Lipid Peroxidation in Brain Tissue

To estimate lipid peroxidation levels, 0.3 mL of the brain tissue homogenate was transferred into an Eppendorf tube and incubated at 37°C for 90 minutes in a metabolic water bath shaker set to 130 strokes per minute. A separate 0.3 mL homogenate sample was stored at 4°C for the incubation process. After two hours, 0.5 mL of 5% trichloroacetic acid (TCA) and 0.5 mL of 0.72% thiobarbituric acid (TBA) were added to the respective samples at their designated temperatures (0°C and 37°C).

The reaction mixtures were then transferred into centrifuge tubes and centrifuged at 3800 × g for 25 minutes. The supernatants were carefully collected into separate tubes and heated in a boiling water bath for 15 minutes. After cooling, the absorbance of the samples was measured at 530 nm using a spectrophotometer (BIOBASE BK-UV1800PC, China). Lipid peroxidation levels were expressed as nanomoles of thiobarbituric acid-reactive substances (TBARS) formed per minute per milligram of lipid.

### 3.7. Estimation of the Content of Glutathione in Brain Tissue

Glutathione (GSH) levels in cerebral tissue were quantified by extracting proteins from the homogenate. An equivalent volume of 10% TCA was added to the homogenate and subsequently centrifuged. To 0.1 mL of the resultant supernatant, 2 mL of phosphate buffer (pH 8.4), 0.5 mL of 5,5′-dithiobis-(2-nitrobenzoic acid), and 0.4 mL of distilled water were added and stirred thoroughly. The solution was vortexed for 15 minutes, and absorbance was quantified at 412 nm using a spectrophotometer (BIOBASE BK-UV1800PC, China). The concentration of reduced GSH was expressed in grams per milligram of protein.

### 3.8. Molecular Docking Study

Molecular docking simulation was carried out against AChE (PDB ID: 4EY7) and BuChE (PDB ID: 2XQI) using mvd 7.0 (Molegro Virtual Docker, Molexus IVS, Denmark). The docking algorithm is based on a grid-based docking methodology, in which the binding pockets and active sites of the protein targets were first located. The active site of AChE was detected at the catalytic triad (Ser203, His447, and Glu334), whereas the active site of BuChE was detected at the catalytic triad (Ser198, His438, and Glu325).

The bond flexibility and side-chain flexibility were set to standard values (tolerance = 1.0, strength = 0.90). The root mean square deviation (RMSD) threshold was set to 2.00 Å, and the number of iterations was set to 1,000. Flexible ligands and protein receptors usually result in a more realistic simulation using MVD, enabling more accurate docking results. Molecular docking was carried out with a minimum of 50 poses and a maximum of 100 runs. The best pose of the compounds was selected as the top hit.

### 3.9. Molecular Dynamics Simulation

Molecular dynamics (MD) simulation for the top docking candidates of the protein-ligand complexes of AChE and BuChE was investigated using Desmond (Schrödinger Inc., USA). The protein preparation wizard was employed to preprocess the protein-ligand complexes. The System Builder tool was used to prepare the systems using the OPLS_2005 force field. A solvent model was applied to simulate physiological conditions, utilizing TIP3P water in an orthorhombic box that was neutralized with counter ions and 0.15 M NaCl. The Martyna-Tuckerman-Klein barostat was used to simulate the NPT ensemble at 300 K and 1 atm pressure. MD simulation was run for 100 ns, and the simulation stability was evaluated by analyzing protein-ligand contacts and calculating RMSD shifts.

### 3.10. ADME-Toxicity Study

An ADME-toxicity study for the top TCM candidates and the control drugs was carried out to assess their pharmacokinetic and safety profiles. ADME-toxicity analysis was conducted using ADMET-AI, which predicts ADMET characteristics through a graph neural network architecture known as Chemprop-RD Kit. The biological activities, ADME characteristics, and toxicity profiles of the hits were assessed. This analysis offers several computational approaches for predicting pharmacokinetic and toxicological characteristics.

### 3.11. Statistical Analysis

All statistical analyses were performed using one-way analysis of variance (ANOVA) to evaluate differences among treatment groups. Tukey’s post-hoc test was applied for pairwise comparisons to identify significant differences between groups. Data are presented as mean ± standard deviation (SD). Statistical significance was set at P < 0.05, with specific significance levels indicated as follows: P < 0.05 (*), P < 0.001 (#). All statistical analyses were conducted using IBM SPSS 21.0 (IBM Corp., NY, and USA).

## 4. Results

### 4.1. In Vitro Cholinesterase Inhibition

Two cholinesterase enzymes, AChE and BuChE, were selected to evaluate the inhibitory activity of the nine TCM compounds. The inhibition was compared with that of donepezil and tacrine. All nine TCM compounds demonstrated strong inhibitory activity in the nanomolar range, with IC₅₀ values ranging from 15.21 ± 2.11 nM to 102.34 ± 3.13 nM against BuChE, and from 33.57 ± 1.91 nM to 90.41 ± 4.41 nM against AChE, as detailed in Figure 2.

**Figure 2.**
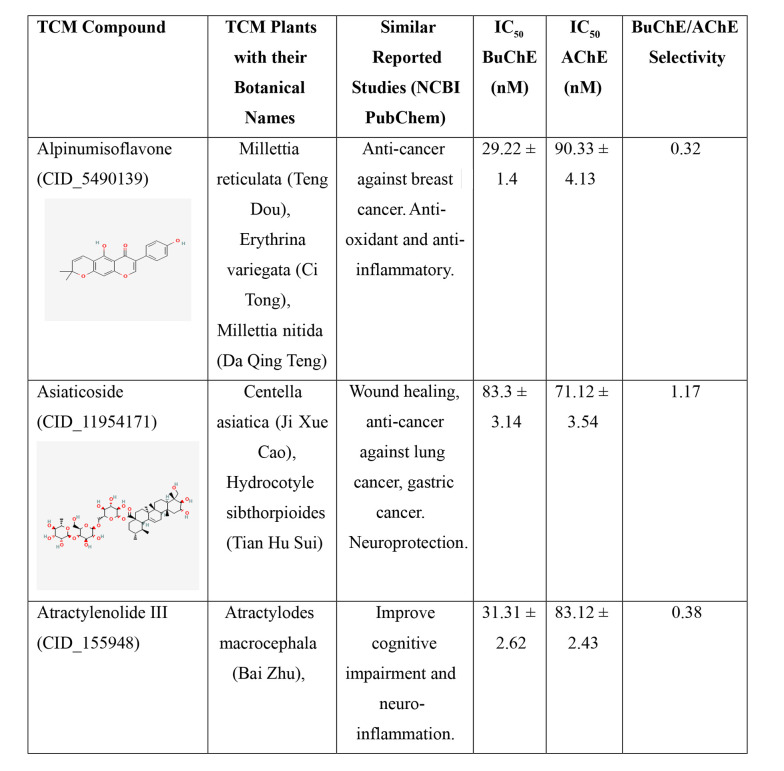
IC_50_ values of the traditional Chinese medicine (TCM) compounds against acetylcholinesterase (AChE) and butyrylcholinesterase (BuChE). IC_50_ values for AChE and BuChE inhibition represents the mean of three independent experiments (n = 3), each performed in triplicate. Data are presented as mean ± standard deviation (SD).

**Figure 2.**
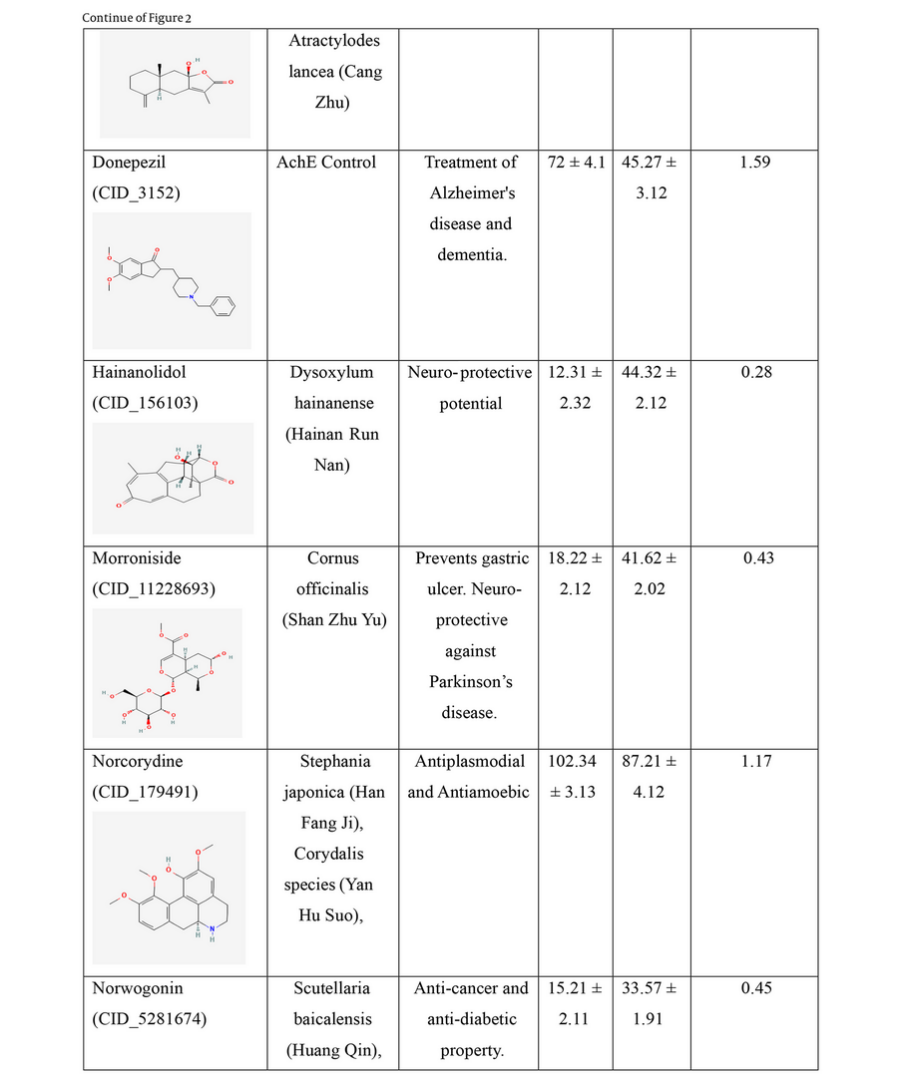
IC_50_ values of the traditional Chinese medicine (TCM) compounds against acetylcholinesterase (AChE) and butyrylcholinesterase (BuChE). IC_50_ values for AChE and BuChE inhibition represents the mean of three independent experiments (n = 3), each performed in triplicate. Data are presented as mean ± standard deviation (SD).

**Figure 2.**
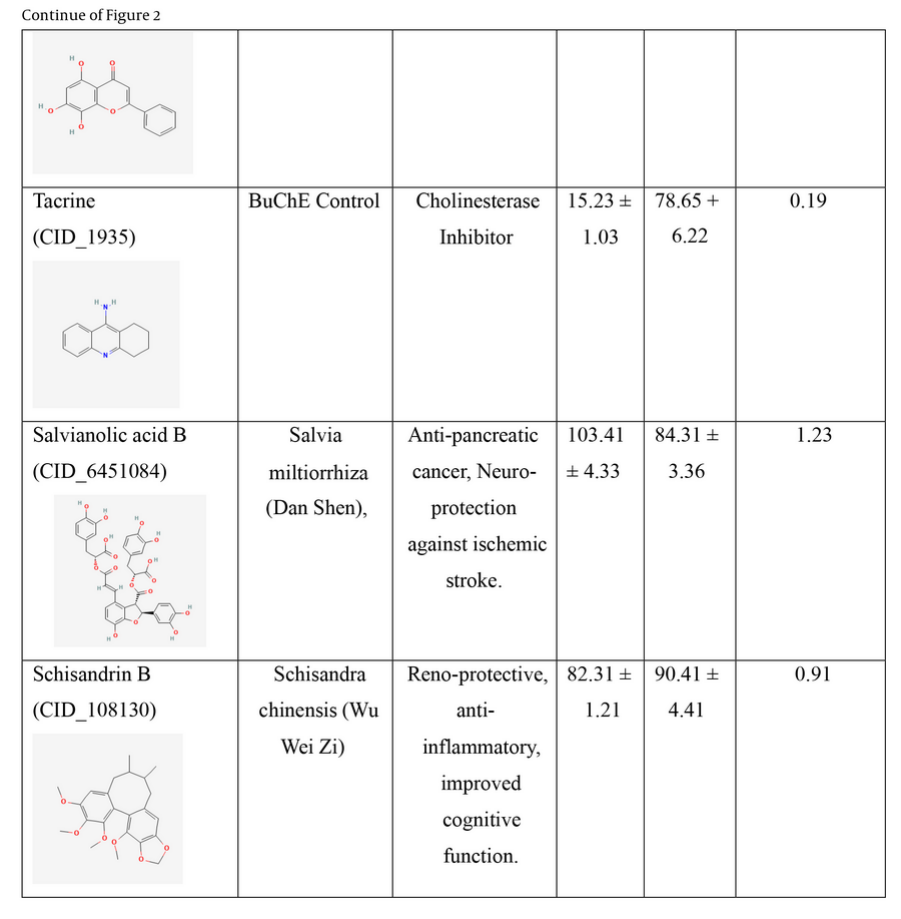
IC_50_ values of the traditional Chinese medicine (TCM) compounds against acetylcholinesterase (AChE) and butyrylcholinesterase (BuChE). IC_50_ values for AChE and BuChE inhibition represents the mean of three independent experiments (n = 3), each performed in triplicate. Data are presented as mean ± standard deviation (SD).

Hainanolidol and norwogonin exhibited superior inhibitory potential against BuChE compared to AChE when assessed against their respective control medicines. In terms of BuChE inhibition, hainanolidol exhibited superior inhibitory action with an IC_50_ value of 12.31 nM, in contrast to the control medication tacrine, which had an IC_50_ value of 15.23 nM. Conversely, the compound norwogonin exhibited superior inhibitory efficacy against AChE, with an IC_50_ value of 33.57 nM, compared to donepezil's 45.27 nM. Hainanolidol demonstrated significant inhibitory efficacy against BuChE and also exhibited notable inhibition of AChE, with an IC_50_ value of 44.32 nM. Norwogonin exhibited a significant inhibitory effect against BuChE, with an IC_50_ value of 15.21 nM.

### 4.2. Lead Acetate-Induced Oxidative Stress in Rat Models

**Figure 3.**
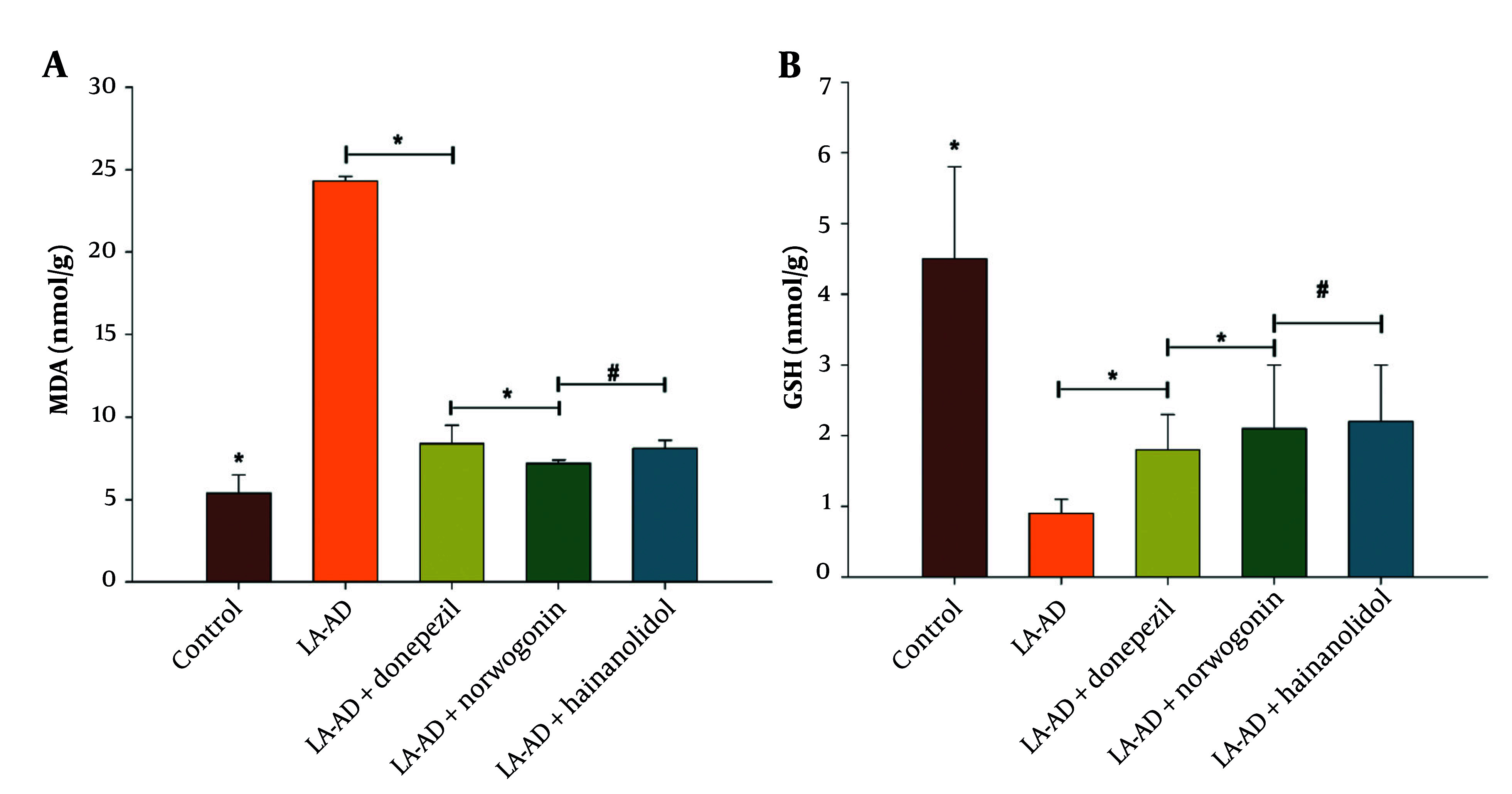
Effect of traditional Chinese medicine (TCM) compounds norwogonin and hainanolidol, compared to the control drug donepezil, on oxidative stress markers in lead acetate-induced AD (LA-AD) rats. A, Malondialdehyde (MDA) levels were measured as an indicator of lipid peroxidation, where lower MDA levels suggest reduced oxidative damage; B, glutathione (GSH) levels were assessed as a marker of antioxidant defense, with higher GSH levels indicating enhanced antioxidant capacity. LA-AD rats were randomly assigned to treatment groups, receiving either norwogonin, hainanolidol, and donepezil (control) for 15 days [data are expressed as mean ± standard deviation (SD), with statistical analysis performed using one-way analysis of variance (ANOVA) followed by Tukey’s post-hoc test. Statistical significance was determined relative to the control group (P < 0.05), with specific significance levels denoted by *, P < 0.05 and #, P < 0.001].

The biological activity of the TCM compounds, namely norwogonin and hainanolidol, was assessed as potent inhibitors of both BuChE and AChE in vivo through a rat model induced with lead acetate to mimic AD. Results showed that the LA-AD group exhibited the highest levels of malondialdehyde (MDA) and the lowest levels of GSH, suggesting the greatest oxidative stress among the groups. Figure 3 showed that MDA levels were significantly reduced, while GSH levels were significantly increased in groups treated with hainanolidol, norwogonin, or donepezil compared to the LA-AD group. Furthermore, both TCM compounds, hainanolidol and norwogonin, effectively normalized MDA levels. Biomarkers of oxidative stress have contributed to elucidating the protective effects of medicinal compounds against neurodegenerative diseases caused by lead acetate.

### 4.3. Evaluation of Acetylcholinesterase and Butyrylcholinesterase Levels

**Figure 4.**
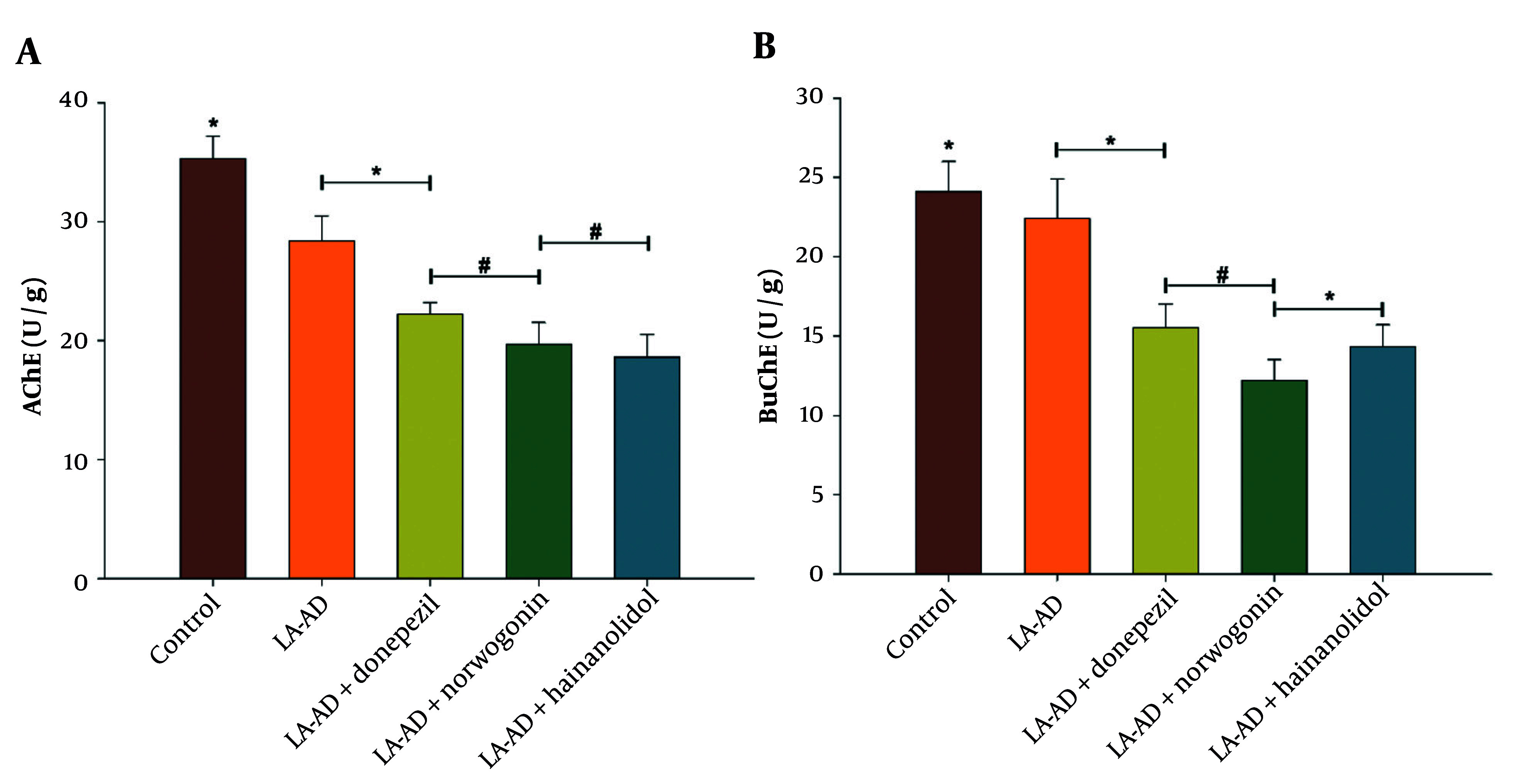
Effect of traditional Chinese medicine (TCM) compounds norwogonin and hainanolidol, compared to the control drug donepezil, on cholinergic enzyme activity in lead acetate-induced AD (LA-AD) rats. A, Acetylcholinesterase (AChE) levels were measured as an indicator of AChE inhibition, with lower AChE levels suggesting improved cholinergic function; B, butyrylcholinesterase (BuChE) levels were assessed to evaluate BuChE inhibition, which is relevant for modulating cholinergic signaling. The LA-AD rats were randomly assigned to treatment groups receiving norwogonin, hainanolidol and donepezil (control) for 15 days [data are expressed as mean ± standard deviation (SD) for each treatment group. Statistical analysis was performed using one-way analysis of variance (ANOVA) followed by Tukey’s post-hoc test. Statistical significance was determined relative to the control group (P < 0.05), with specific significance levels denoted by *, P < 0.05 and #, P < 0.001].

Further, the study examined the effects of hainanolidol and norwogonin on AChE and BuChE activities in rats. While hainanolidol and norwogonin treatments significantly decreased the activities of AChE and BuChE, the control group and the LA-AD group showed very little decrease in the activities of these enzymes, with only slight reductions observed (Figure 4). 

The AChE activity levels in the groups treated with these two compounds were significantly lower than those in the donepezil group, and the lower levels suggested that these compounds could be more potent in inhibiting AChE activity than the widely used cholinesterase inhibitor donepezil. According to the study, hainanolidol and norwogonin might be more successful in lowering brain concentrations of BuChE compared to AChE, making them potential therapeutic agents for AD, possibly acting more expeditiously than the already approved cholinesterase inhibitor Rivastigmine.

### 4.4. Molecular Docking Study

Table 1 shows the docking results of the nine TCM compounds at the active site of AChE. The study analyzed the binding affinity of two TCM compounds, norwogonin and hainanolidol, to AChE. The results showed that norwogonin and hainanolidol exhibited greater binding affinity, making them potential therapeutic candidates for AD. Hainanolidol scored most effectively with a MolDock Score of -305.21 kcal/mol.

**Table 1.** Docking Results of Traditional Chinese Medicine Compounds at the Active Site of Acetylcholinesterase (AChE, PDB ID: 4EY7), Compared with Donepezil as a Control ^a^

Compound	MolDock Score	Rerank Score	Interaction Energy	HBond	Binding Affinit	Total Score
**Norwogonin**	-149.73	-120.5	-93.21	-5.34	-23.32	-392.1
**Hainanolidol**	-136.2	-110.63	-86.32	-9.93	-21.32	-364.4
**Donepezil (control)**	-135.83	-121.62	-83.32	-2.89	-20.21	-363.87
**Morroniside**	-121.5	-111.27	-73.21	-5.14	-19.21	-330.33
**Norcorydine**	-117.56	-109.47	-68.42	-3.49	-20.21	-319.15
**Atractylenolide III**	-116.32	-110.85	-70.12	0	-12.21	-309.5
**Rivastigmine**	-119.16	-95.49	-69.32	-4.76	-18.21	-306.94
**Schisandrin B**	-110.51	-81.71	-76.13	0.1	-14.21	-282.46
**Alpinumisoflavone**	-106.94	-76.81	-74.52	0.31	-16.63	-274.59
**Salvianolic acid B**	-102.41	-71.78	-69.31	-3.34	-21.31	-268.15
**Asiaticoside**	-89.79	-71.62	-63.32	3.28	-22.31	-243.76

^a^ Values are expressed as kcal/mol.

**Figure 5.**
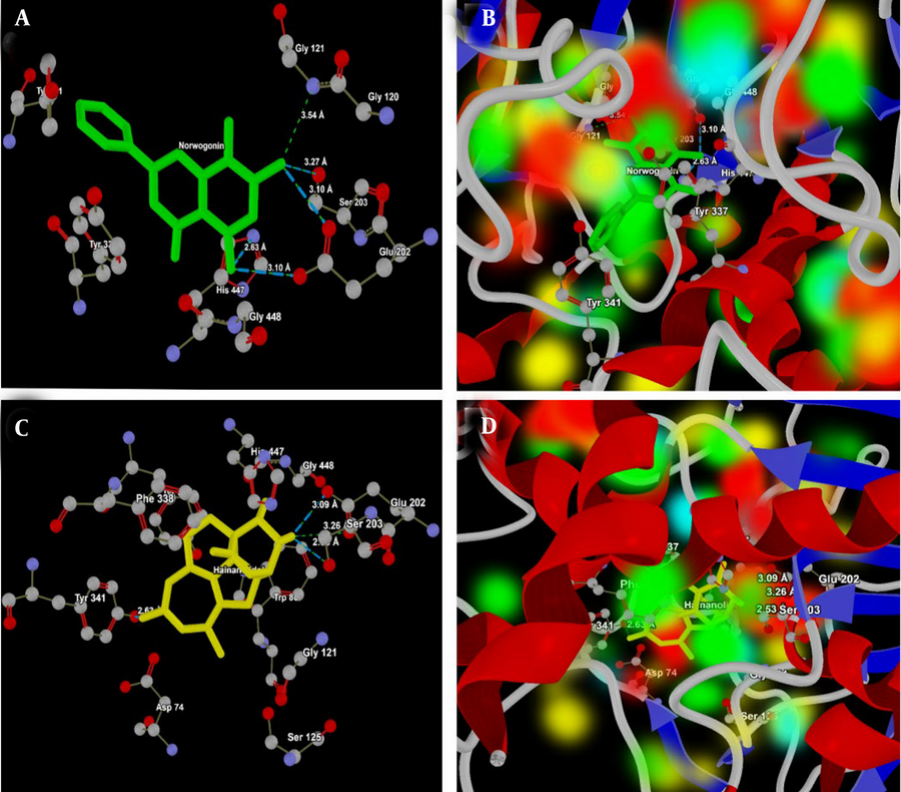
A, Interaction analysis of norwogonin (green) with the active site of acetylcholinesterase (AChE, PDB ID: 4EY7). The ligand forms strong hydrogen bonds with the catalytic triad residues His447 (NE2), Ser 203 (OG), Glu121 (N) and Glu202 (OE1 and OE2), highlighting its potential as a selective inhibitor of AChE; B, energy map representation of norwogonin at the active site of AChE, illustrating hydrogen bond acceptor (HBA) and donor (HBD) regions along with electropositive and electronegative areas; C, interaction analysis of hainanolidol (yellow) with the active site of AChE. The ligand interacts strongly with the catalytic residue Ser203 (OE1, OE2 and OG) and the peripheral anionic site residue Tyr341 (OH) suggesting effective AChE inhibition; D, energy map representation of hainanolidol at the active site of AChE, highlighting HBA/HBD regions and electrostatic complementarity.

Hainanolidol and norwogonin, which formed strong hydrogen bonds, appear promising as BuChE inhibitors, with docking results superior to those of tacrine and donepezil, potentially offering new therapeutic options for treating AD or other neurodegenerative disorders (Table 2). The molecular interactions of norwogonin and hainanolidol with AChE (PDB ID: 4EY7) are presented in Table 3. Norwogonin shows a more focused interaction profile, mainly targeting the catalytic triad residues (Ser203, His447, and Glu202) (Figure 5A), which indicates it could act as a selective inhibitor.

**Table 2.** Docking Results of Traditional Chinese Medicine Compounds at the Active Site of Butyrylcholinesterase (BuChE, PDB ID: 2XQI), Compared with Tacrine as a Control ^a^

Compound	MolDock Score	Rerank Score	Interaction Energy	HBond	Binding Affinity	Total Score
**Hainanolidol**	-98.01	-72.50	-91.21	-19.041	-24.45	-305.21
**Norwogonin **	-94.82	-61.05	-86.53	-9.157	-28.32	-279.88
**Tacrine (control)**	-94.30	-69.54	-71.42	-6.752	-21.32	-263.34
**Alpinumisoflavone**	-69.99	-78.56	-82.42	-0.683	-21.32	-252.98
**Morroniside**	-73.12	-69.05	-80.18	-3.714	-20.21	-246.27
**Donepezil**	-69.70	-63.65	-79.32	-1.234	-18.53	-232.43
**Norcorydine**	-78.42	-70.61	-68.43	0	-14.32	-231.78
**Schisandrin B**	-80.46	-65.14	-65.12	-2.215	-18.32	-231.25
**Salvianolic acid B**	-75.82	-67.47	-68.43	-9.488	-10.11	-231.32
**Atractylenolide III**	-71.21	-70.12	-62.43	0	-12.21	-215.97
**Asiaticoside**	-48.70	-68.10	-64.23	-7.889	-8.12	-197.03

^a^ Values are expressed as kcal/mol.

**Table 3.** Molecular Interaction Analysis of Hainanolidol and Norwogonin at the Active Site of Acetylcholinesterase (AChE, PDB ID: 4EY7) ^a^

Compounds and Ligand-Protein Interactions	Site	Interaction Distance (Å)	Interaction Energy (kcal/mol)
**Hainanolidol**			
O(1)---OE1 (Ser203)	Catalytic triad	3.26	-0.37
O(1)---OE2(Ser203)	Catalytic triad	3.09	-2.5
O(1)---OG (Ser203)	Catalytic triad	2.53	-2.0
O(3)---OH (Tyr341)	Peripheral anionic site	2.63	-2.5
**Norwogonin**			
O(3)---N(Glu121)	Oxyanion hole	2.54	-0.5
O(3)---OE1(Glu202)	Peripheral	3.10	-2.5
O(3)---OG(Ser203)	Catalytic triad	3.27	1.65
O(1)---NE2(His447)	Catalytic triad	2.63	-2.5
O(1)---OE2 (Glu202)	Catalytic site	3.10	-2.5

^a^ The table presents detailed ligand-protein interactions, highlighting the specific residues involved, interaction distances, and interaction energies.

Figure 5B presents the energy map of the 4EY7 active site, which shows the electropositive and electronegative areas of Norwogonin in the HBA/HBD regions, as well as the HBA/HBD regions of the 4EY7 active site. However, hainanolidol forms multiple bonds with the key catalytic residue Ser203, as well as Tyr341, a residue in the peripheral anionic site, demonstrating its strong ability to inhibit AChE activity (Figure 5C). At the active site of 4EY7, the HBA/HBD and electropositive/negative regions of hainanolidol are shown in the energy map (Figure 5D), and the oxygen atoms of Ser203, OE1, OE2, and OG interact with hainanolidol, with distances varying from 2.53 Å to 3.26 Å.

 Although both hainanolidol and norwogonin interact partially with catalytic and partially with peripheral sites, distinctive differences in interactions with the catalytic residues suggest that norwogonin might act more selectively as an AChE inhibitor. Table 4 presents a detailed analysis of the ligand-protein interactions between hainanolidol, norwogonin, and key amino acid residues at the active site of BuChE (PDB ID: 2XQI).

**Table 4.** Molecular Interaction Analysis of Hainanolidol and Norwogonin at the Active Site of Butyrylcholinesterase (BuChE, PDB ID: 2XQI) ^a^

Compounds and Ligand-Protein Interactions	Site	Interaction Distance (Å)	Interaction Energy (kcal/mol)
**Hainanolidol**			
O(1)---OG (Ser198)	Catalytic triad	3.23	-1.8
O(1)--- NE2(His438)	Catalytic triad	2.82	-2.5
O(1) ---OE1(Glu197)	Catalytic site	3.10	-1.76
O(1)---OE2(Glu197)	Catalytic site	3.10	-1.72
O(3)---OH(Tyr332)	Peripheral anionic site	3.45	-0.05
**Norwogonin**			
O(3)---OE1(Glu197)	Catalytic site	2.61	-2.5
O(2)---N7(Glu197)	Catalytic site	2.76	-2.5
O(2)---OG(Ser198)	Catalytic triad	2.87	-2.5
O(2)---N7(His438)	Catalytic triad	3.10	-2.5

^a^ The table presents detailed ligand-protein interactions, highlighting the specific residues involved, interaction distances, and interaction energies.

**Figure 6.**
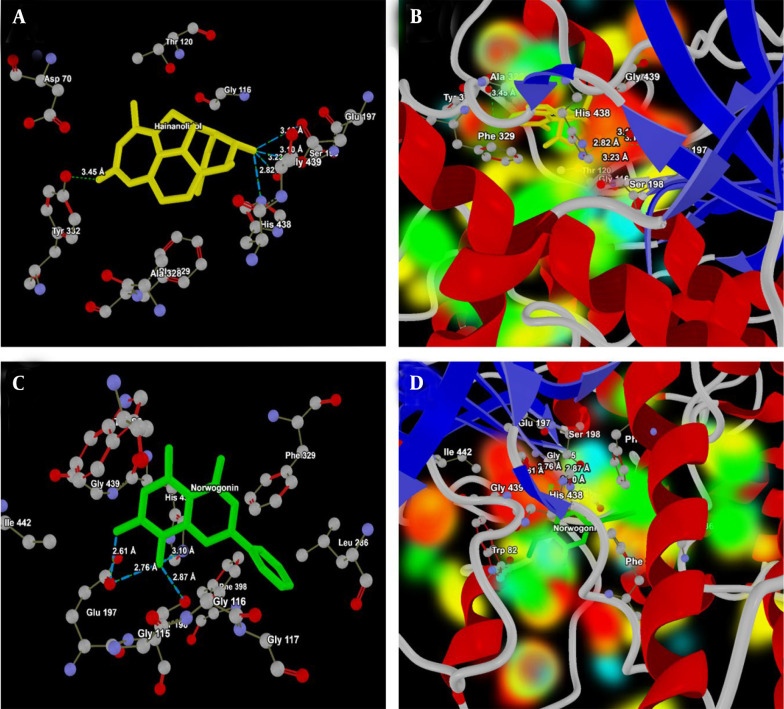
A, Interaction analysis of hainanolidol (yellow) with the active site of butylcholinesterase (BuChE, PDB ID: 2XQI); the ligand forms strong hydrogen bonds with the catalytic triad residues Glu197 (OE2 and OH), Ser 198 (OG) and His438 (NE2), highlighting its potential as a selective inhibitor of BuChE; B, energy map representation of hainanolidol at the active site of BuChE, illustrating hydrogen bond acceptor (HBA) and donor (HBD) regions along with electropositive and electronegative areas; C, interaction analysis of norwogonin (green) with the active site of BuChEl. The ligand interacts strongly with the catalytic triad Ser198 (OG), His438 (N7) and catalytic site Glu197 (OE1, and N7) suggesting effective BuChE inhibition; D, energy map representation of norwogonin at the active site of BuChE, highlighting HBA/HBD regions and electrostatic complementarity.

Because these compounds form strong binding interactions, their potential effectiveness is closely related to their interaction patterns with the target molecule, particularly hydrogen bonding. The BuChE active site interacts with hainanolidol at several key residues: Ser198, His438, Glu197 (part of the catalytic triad), and Tyr332 (Figure 6A). The way hainanolidol interacts with the catalytic triad suggests that hainanolidol binds strongly and could be a potent BuChE inhibitor. The energy map of hainanolidol at the 2XQI active site, showing the HBA/HBD regions and electropositive/negative areas, is presented in Figure 6B. As shown in Figure 6C, norwogonin further interacts with Glu197, Ser198, and His438. This illustrates its potential as a BuChE inhibitor due to its strong interactions within the catalytic triad of the ligand-protein complex. The energy map analysis (Figure 6D) shows the HBA/HBD regions and electropositive/negative areas of hainanolidol at the 2XQI active site. Both hainanolidol and norwogonin form strong binding interactions at the BuChE active site, particularly with the catalytic triad. While hainanolidol’s diverse interaction profile might contribute to its stability, norwogonin’s tight interactions, especially with Glu197, might enhance its inhibitory potential.

### 4.5. Molecular Dynamics Simulation Analysis

**Figure 7.**
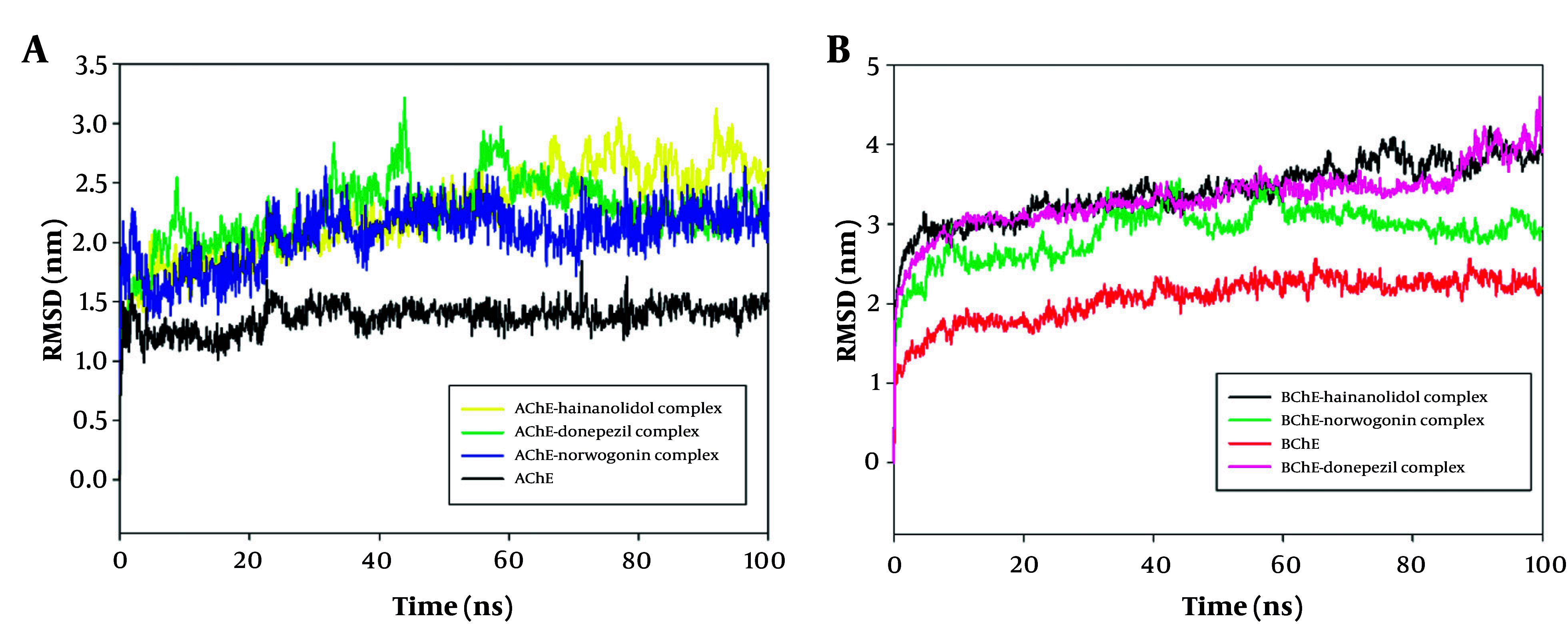
A, Root mean square deviation (RMSD) analysis of norwogonin and hainanolidol complexed with acetylcholinesterase (AChE, PDB ID: 4EY7) during a 100 ns molecular dynamics (MD) simulation. The RMSD profiles reveal minimal fluctuations, indicating early equilibrium and sustained structural stability of the protein-ligand complexes throughout the simulation period; B, RMSD analysis of norwogonin and hainanolidol complexed with butyrylcholinesterase (BuChe, PDB ID: 2XQI) during a 100 ns MD simulation. The trajectories show steady RMSD values after the initial equilibration phase, confirming robust ligand binding and stable conformations of the protein-ligand complexes.

The 100 ns MD simulations were performed on AChE and BuChE complexes with the ligands norwogonin and hainanolidol to determine their structural stability and binding characteristics. The RMSD analysis presented in Figure 7 observes the conformational stability of these protein-ligand complexes over the course of the simulation. The RMSD profile shows, with little deviation, that the norwogonin and hainanolidol complexes with AChE remained stable under the given conditions throughout the 100 ns simulation. Early in the simulation, both complexes achieved equilibration, indicating that ligand binding and protein conformation remained stable (Figure 7A). Interestingly, for BuChE, the RMSD trajectories for each complex showed consistent progressive stability even after the initial equilibration phase, further indicating a very solid binding of these ligands to the receptor (Figure 7B). Therefore, norwogonin and hainanolidol demonstrated favorable interactions with both AChE and BuChE, thereby contributing to higher inhibition against their enzyme activities.

### 4.6. ADME-Toxicity Analysis

**Figure 8.**
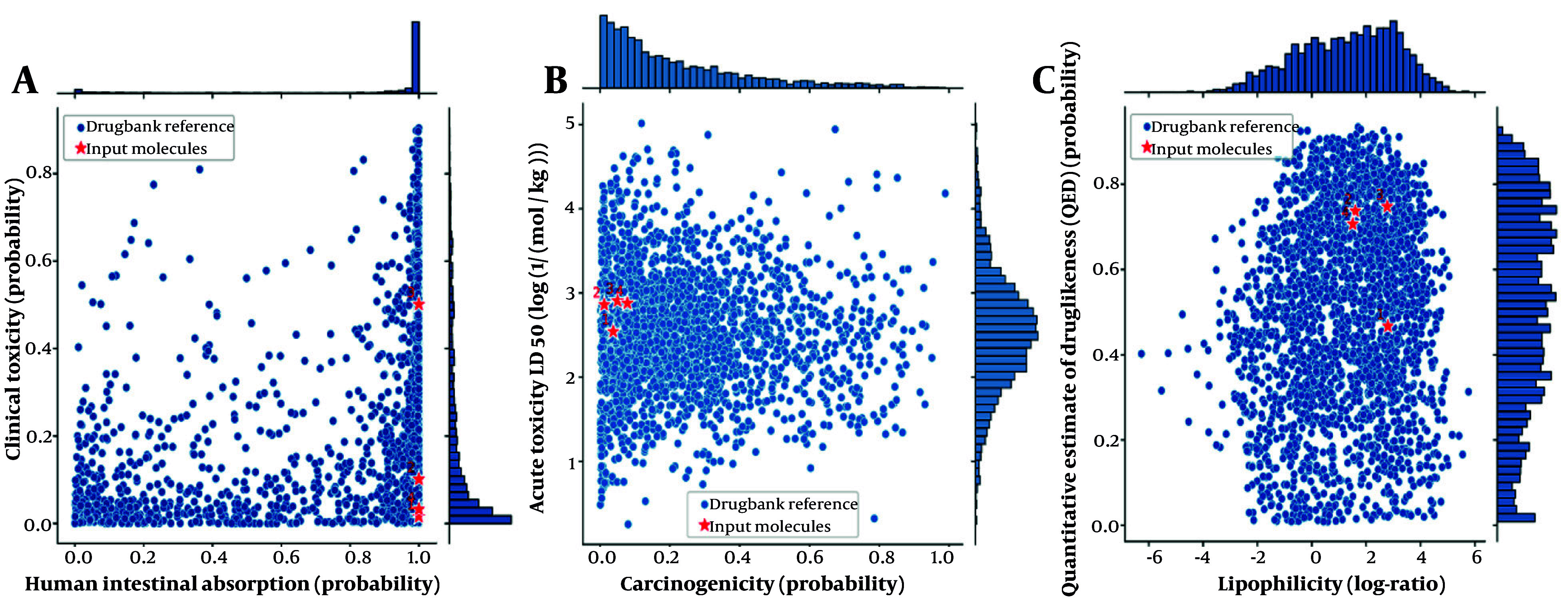
A, Human intestinal absorption versus clinical toxicity plot for norwogonin and hainanolidol. Both compounds fall within the high absorption and low toxicity region, indicating suitability for oral delivery and minimal risk of adverse effects; B, clinical toxicity versus acute toxicity (LD_50_) plot. The compounds demonstrate a balance between low acute toxicity and clinical safety, highlighting their therapeutic potential; C, carcinogenicity versus acute toxicity (LD_50_) plot. Both norwogonin and hainanolidol are within the non-carcinogenic range, consistent with safety benchmarks for FDA-approved drugs. Compound legends: (1) Norwogonin, (2) hainanolidol, (3) donepezil, and (4) tacrine.

The ADME-ToX (absorption, distribution, metabolism, excretion, and toxicity) analysis provides a comprehensive evaluation of the pharmacokinetic and toxicological properties of both norwogonin and hainanolidol compared to drug candidates and FDA-approved drugs for AD. Both norwogonin and hainanolidol exhibited high absorption potential, indicating suitability for oral delivery. Furthermore, they fall within the non-toxic range, suggesting they are unlikely to produce adverse clinical effects when administered at therapeutic doses (Figure 8A). Figure 8B shows that norwogonin and hainanolidol demonstrated acute low toxicity while remaining safe for clinical use, thereby reinforcing their therapeutic potential. Both compounds fall within the non-carcinogenic range according to FDA-approved drug benchmarks. Their very high LD_50_ values further confirm their acute safe exposure profile (Figure 8C). 

## 5. Discussion

Traditional Chinese medicine has shown great potential in the treatment of AD, which is characterized by Aβ plaques, NFTs of hyperphosphorylated tau protein, and progressive neuronal loss. The involvement of enzymes such as AChE and BuChE in the pathogenesis of AD makes these two important therapeutic targets (11). The present study aims to investigate the in vitro and in vivo inhibitory potential of nine TCM compounds on AChE and BuChE enzymes in order to explore their potential neuroprotective effects.

A total of nine TCM compounds were evaluated alongside the well-established anti-Alzheimer’s drugs donepezil and tacrine (12). The in vitro results indicated that out of the nine compounds, norwogonin and hainanolidol displayed very strong inhibition of AChE and BuChE. The IC_50_ values for norwogonin were 15.21 ± 2.11 nM for BuChE and 33.57 ± 1.91 nM for AChE, showing potent inhibition. Similarly, hainanolidol exhibited IC_50_ values of 12.31 ± 2.32 nM for BuChE and 44.32 ± 2.12 nM for AChE, both demonstrating potent inhibition comparable to the reference drugs donepezil and tacrine. Hainanolidol showed stronger inhibition of BuChE with an IC_50_ of 12.31 nM, which was better than tacrine (15.23 nM). In contrast, norwogonin was more effective against AChE, with an IC_50_ of 33.57 nM compared to 45.27 nM for donepezil. These findings suggest that both TCM compounds could serve as potential therapeutic adjuvants for AD due to their balanced inhibition of AChE and BuChE.

Both norwogonin and hainanolidol were also shown to possess neuroprotective effects using the LA-AD rat model. Treatment with these compounds resulted in decreases in MDA levels and increases in GSH levels, which are indicators of oxidative stress. These results are consistent with studies that have found that antioxidant compounds reduce oxidative damage in the brain, an important hallmark of AD pathology (13). These findings indicate that both TCM compounds possess antioxidative properties and may have potential for protecting neurons from oxidative damage, which is believed to contribute to AD-induced neurodegeneration. In the present study, hainanolidol and norwogonin demonstrated significant antioxidant effects by reducing oxidative stress induced by apoptosis in brain tissue, specifically through decreasing MDA levels and increasing GSH content.

These results are consistent with reported neuroprotective actions of compounds that reduce oxidative damage induced in neurocytes by PbAc (14). Furthermore, the present study’s findings align with a number of recent studies demonstrating a significant correlation between neuroprotective action and the amelioration of the brain's oxidative status through reductions in MDA levels and enhancements in GSH levels (15).

While the study demonstrates promising in vitro and in vivo findings, the direct clinical relevance of norwogonin and hainanolidol remains speculative due to the absence of human and translational data, which is a limitation of the present study. Although the results suggest potent cholinesterase inhibition and neuroprotection in a LA-AD rat model, extrapolation to human AD pathology requires caution. Future studies should incorporate pharmacokinetic profiling in humans, blood-brain barrier permeability assessments, and clinical trials to validate their therapeutic potential (16). In fact, oxidative stress is a major contributor to AD pathology, leading to neuronal apoptosis and synaptic dysfunction (17). The observed reduction in MDA levels and increase in GSH levels suggest that these compounds may modulate redox homeostasis. Possible mechanisms include activation of the Nrf2/ARE pathway, which regulates antioxidant enzyme expression (18), and direct free radical scavenging (19). Additionally, the interaction of norwogonin and hainanolidol with key mitochondrial proteins involved in oxidative phosphorylation should be explored, as mitochondrial dysfunction is a major source of reactive oxygen species (ROS) in AD (20).

To further validate their antioxidant properties, future studies should assess their effects on ROS qproduction, mitochondrial membrane potential, and apoptosis-related pathways, such as caspase activation and Bcl-2/Bax expression ratios (21). Incorporating multi-omics approaches, such as transcriptomics or metabolomics, may also provide deeper insights into their molecular mechanisms in neurodegeneration (22). Furthermore, cholinesterase activity in the rat brain showed that both hainanolidol and norwogonin significantly reduced the activities of AChE and BuChE. Compared to the donepezil-treated group, these compounds reduced AChE levels more effectively. This finding aligns with the results from the in vitro assays and highlights their potential as dual inhibitors of both AChE and BuChE. Such inhibition is a critical mechanism in AD management, as increasing acetylcholine levels in the brain is associated with improvements in cognitive function (23).

Further, the molecular docking analysis provided additional insights into the binding affinities of hainanolidol and norwogonin at the active sites of AChE and BuChE. Both compounds showed favorable docking scores at the AChE and BuChE active sites, indicating strong binding interactions. Norwogonin exhibited the lowest MolDock Score and Interaction Energy for AChE, suggesting a high binding affinity (24). The docking results indicated that norwogonin may interact more specifically with the catalytic triad of AChE, which is critical for its inhibitory action. Conversely, hainanolidol demonstrated strong binding interactions with both AChE and BuChE, forming multiple hydrogen bonds. Among these, the strong interactions of hainanolidol with Ser203 in AChE and Ser198 in BuChE are most notable, implying its dual inhibitory activity on both AChE and BuChE enzymes (25).

The MD simulations demonstrated structural stability and strong binding interactions between norwogonin and hainanolidol with the target proteins AChE and BuChE. RMSD analysis confirmed the robustness of the protein-ligand complexes, showing stable trajectories throughout the simulation. Conformational stability was validated by consistent RMSD shifts and favorable interaction dynamics (26). These findings are in accordance with other MD simulation studies, including the study of flavonoid binding to AChE by Azmal et al., 2024, and the study of natural BuChE inhibitors by Nour et al., 2025, reinforcing the role of stable protein-ligand interactions in drug design (27, 28).

Strong and favorable interactions between the ligands and AChE and BuChE were revealed by simulations, highlighting their potential to inhibit enzyme activities. Further understanding will involve detailed analyses of hydrogen bond formation and interaction energy profiles (29), which are crucial for stability and therapeutic efficacy. The ADME-Toxicity analysis also provided pharmacokinetic and toxicological profiles of norwogonin and hainanolidol, showing high human intestinal absorption, efficient systemic absorption, therapeutic potential, and low clinical toxicity (30). These properties meet the standards of safety required for therapeutic applications. Other natural compounds, such as those studied by Wu et al. in polyphenols and by Daoud et al. in terpenoids, have shown similar findings in ADME-Toxicity evaluations, where compounds exhibited drug-like properties and low toxicity in computational assessments (31, 32). The analysis of these two compounds based on their human intestinal absorption vs. clinical toxicity, clinical toxicity vs. acute toxicity, and carcinogenicity vs. acute toxicity plots showed minimized long-term accumulation and toxicity (33). Therefore, the study suggests that norwogonin and hainanolidol are potential therapeutic agents for AD due to their potent cholinesterase inhibitory activities and antioxidative properties. They reduce oxidative stress in the brain and inhibit both AChE and BuChE (34). Moreover, favorable docking scores and interaction profiles of these compounds with both AChE and BuChE support their potential use as lead candidates for AD drug development. The results also highlight the growing consideration of TCM as a valuable source of novel therapeutic agents for neurodegenerative diseases. The compounds demonstrated promising results both in vitro and in vivo and would benefit from further clinical and preclinical studies to confirm their efficacy and safety in human models (35). However, comprehensive assessment of the pharmacokinetics, bioavailability, and long-term effects of these compounds in clinical settings would be the next step in fully evaluating their potential as AD therapeutics.

### 5.1. Conclusions

The study demonstrates that norwogonin and hainanolidol are effective as acute inhibitors of AChE and BuChE — enzymes associated with AD. In addition, they exhibit neuroprotective effects in rat models. Molecular docking and MD simulations further support their therapeutic potential in AD and other neurodegenerative disorders, as confirmed by the findings of this study.

## Data Availability

The dataset presented in the study is available on request from the corresponding author during submission or after publication.
